# Neuroprognostication of Consciousness Recovery in a Patient with COVID-19 Related Encephalitis: Preliminary Findings from a Multimodal Approach

**DOI:** 10.3390/brainsci10110845

**Published:** 2020-11-12

**Authors:** Aude Sangare, Anceline Dong, Melanie Valente, Nadya Pyatigorskaya, Albert Cao, Victor Altmayer, Julie Zyss, Virginie Lambrecq, Damien Roux, Quentin Morlon, Pauline Perez, Amina Ben Salah, Sara Virolle, Louis Puybasset, Jacobo D Sitt, Benjamin Rohaut, Lionel Naccache

**Affiliations:** 1 Brain institute—ICM, Inserm U1127, CNRS UMR 7225, Sorbonne Université, 75013 Paris, France; melanie.denisvalente@icm-institute.org (M.V.); nadya.pyatigorskaya@icm-institute.org (N.P.); virginie.lambrecq@aphp.fr (V.L.); pauline.perez@icm-institute.org (P.P.); amina.bensalah@icm-institute.org (A.B.S.); jacobo.sitt@icm-institute.org (J.D.S.); benjamin.rohaut@icm-institute.org (B.R.); lionel.naccache@upmc.fr (L.N.); 2 CNRS, INSERM, Laboratoire d’Imagerie Biomédicale, Sorbonne Université, 75006 Paris, France; albert.cao@aphp.fr (A.C.); louis.puybasset@aphp.fr (L.P.); 3 Department of Neurophysiology, AP-HP, Hôpital Pitié-Salpêtrière, Sorbonne Université, 75006 Paris, France; julie.zyss@aphp.fr; 4 Department of Neurology, Neuro-ICU, AP-HP, Hôpital Pitié-Salpêtrière, Sorbonne Université, 75006 Paris, France; anceline.dong@aphp.fr (A.D.); victor.altmayer@aphp.fr (V.A.); 5 Department of Neuroradiology, AP-HP, Hôpital Pitié-Salpêtrière, Sorbonne Université, 75006 Paris, France; 6 Department of Critical Care, Hôpital Louis Mourier, AP-HP, Université de Paris, 92700 Colombes, France; damien.roux@aphp.fr (D.R.); quentin.morlon@aphp.fr (Q.M.); 7 Department of Pneumology, post ICU rehabilitation, AP-HP, Hôpital Pitié-Salpêtrière, Sorbonne Université, 75006 Paris, France; sara.virolle@aphp.fr; 8 Department of Anesthesiology & Critical Care, AP-HP, Hôpital Pitié-Salpêtrière, Sorbonne Université, 75006 Paris, France; 9 Department of Neurology, Columbia University, New York, NY 10027, USA

**Keywords:** disorders of consciousness, COVID-19, neuroprognostication, EEG, SARS-CoV-2

## Abstract

Predicting the functional recovery of patients with severe neurological condition due to coronavirus disease 2019 (COVID-19) is a challenging task. Only limited outcome data are available, the pathophysiology is poorly understood, and the time-course of recovery is still largely unknown. Here, we report the case of a patient with COVID-19 associated encephalitis presenting as a prolonged state of unresponsiveness for two months, who finally fully recovered consciousness, functional communication, and autonomy after immunotherapy. In a multimodal approach, a high-density resting state EEG revealed a rich brain activity in spite of a severe clinical presentation. Using our previously validated algorithms, we could predict a possible improvement of consciousness in this patient. This case report illustrates the value of a multimodal approach capitalizing on advanced brain-imaging and bedside electrophysiology techniques to improve prognosis accuracy in this complex and new aetiology.

## 1. Introduction

Providing an accurate neurologic prognosis of patients with severe coronavirus disease 2019 (COVID-19) who survived critical illness and experienced a prolonged disorder of consciousness (DOC) is a challenging task. The pathophysiology of SARS-CoV-2 neuropathogenicity is still unknown and long-term prognostic studies remain lacking. More specifically, the impairment of consciousness in patients with COVID-19 is frequently multifactorial in a context that typically combines sepsis, severe hypoxemia, multiorgan failure, intensive care unit (ICU) complication, and toxic or metabolic encephalopathy. A more direct causal effect of SARS-CoV2 on consciousness and cognition has been proposed within the recent frameworks of encephalitis and encephalopathy related to COVID-19 [1,2,3,4,5,6,7,8,9].

Within this highly uncertain context, here, we aimed at applying the multimodal approach developed to improve diagnostic accuracy and prognosis performance in patients suffering from DOC irrespective of the aetiology [10,11]. The combination of repeated behavioral assessment, advanced structural and functional neuroimaging, and electrophysiology techniques are used on a regular basis in several expert DOC centers [12,13,14]. We report here the case of a patient who remained unresponsive for two months after COVID-19 related encephalitis, and who eventually fully recovered after immunotherapy, and was discharged home with a good functional recovery at 5.5 months. This case report illustrates the potentially high relevance of this multi-modal evaluation to improve decision-making in this complex aetiology in which both the possibility of recovery and its time-course remain largely unknown. 

## 2. Clinical Presentation

A hypertensive 56-year-old man presented to the emergency department with fever, dry cough, and dyspnea. He was diagnosed as positive to SARS-Cov2-PCR on a nasopharyngeal swab test. A chest CT scan revealed multiple peripheral patchy ground-glass opacities with typical COVID-19 distribution. Six days after the onset of symptoms, he was admitted in ICU, sedated, and mechanically ventilated for an acute respiratory distress syndrome. The patient then presented several complications including: (i) at day 13 and at day 31, ventilator-associated pneumonias treated successively by third-generation cephalosporin plus linezolide, trimethoprime-sulfamethoxazole, and by meropenem and aminosid, (ii) at day 1, a reversible acute kidney failure that required haemodialysis from day 15 to day 52, (iii) and a multifactorial anemia. Sedation was weaned at day 36, but the patient remained in a vegetative state that is also coined unresponsive wakefulness syndrome (VS/UWS) (Glasgow coma scale = 4/15 (E2-V1-M1), with oculomotor disturbances, skew deviation, and left internuclear ophthalmoplegia. Cerebrospinal fluid (CSF) examination at day 43 showed 1 cell/mm^3^, with normal levels of protein and glucose.

**Figure 1:**
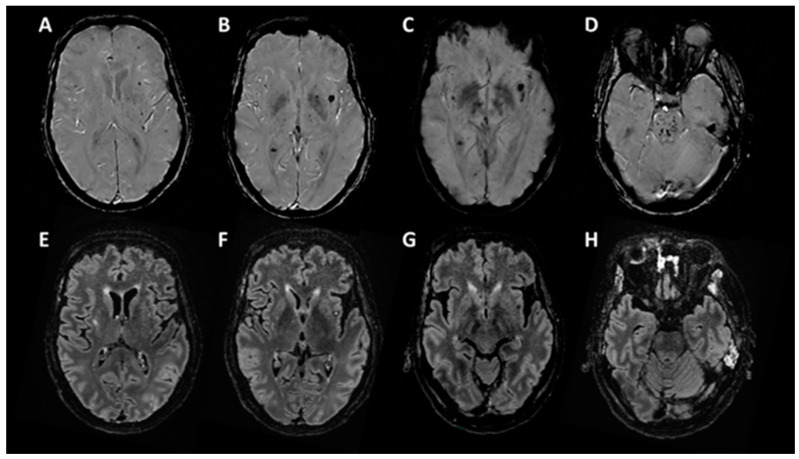
Brain MRI, T2 Star Susceptibility Weighted ANgiography (SWAN) sequence (**A**–**D**), and FLAIR sequence (**E**–**H**).

At day 55, clinically the patient still unresponsive (Glasgow coma scale = 4/15 (E2-V1-M1), Full outline of unresponsiveness (FOUR) score = 6 (E1-M0-B4-R1), coma recovery scale revised (CRS-r) = 2/23 (0-1-0-0-0-1)). A brain MRI at day 55 (Figure 1) showed multiple small haemorrhagic lesions in the pontine tegmentum (D) and the left and right subinsular regions that notably include claustrums, as well as deep ganglia, corpus callosum, and cortico-subcortical regions (A–C). There were few signal abnormalities on FLAIR (E–H) and no associated contrast enhancement. 

**Figure 2:**
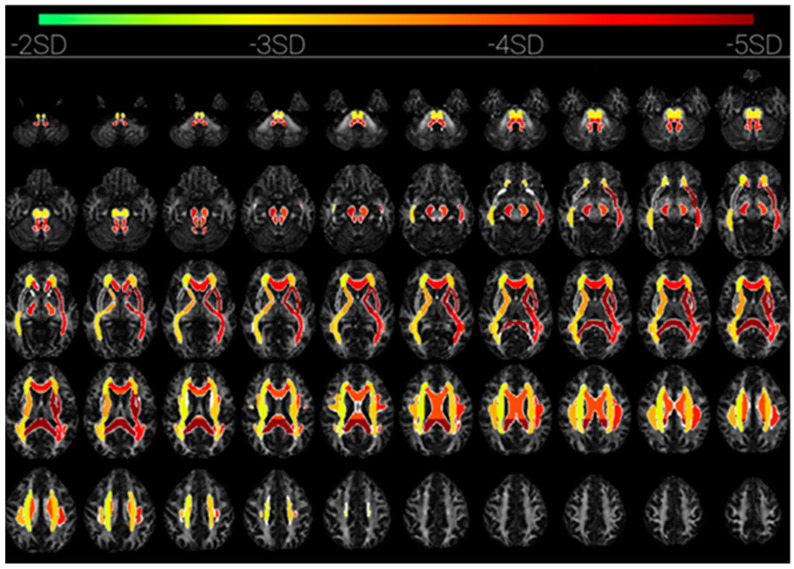
Brain MRI, Diffusion Tensor Imaging DTI sequence: mean fractional anisotropy within regions of interest expressed as number of standard deviations of the normal measurement in healthy subjects (software version 1.1, from brainQuant, Paris, France) [15].

The diffusion tensor imaging revealed a decreased fractional anisotropy (FA) affecting widespread white matter tracts in both hemispheres (software version 1.1, from brainQuant, Paris, France). The FA decrease was asymmetrical and predominated in the left hemisphere, in particular in corona radiata, superior longitudinal fasciculus, anterior and posterior limb of internal capsule, and external capsule. The FA was also decreased in both left and right cerebral pedunculi, and the whole corpus callosum (Figure 2) [15].

**Figure 3:**
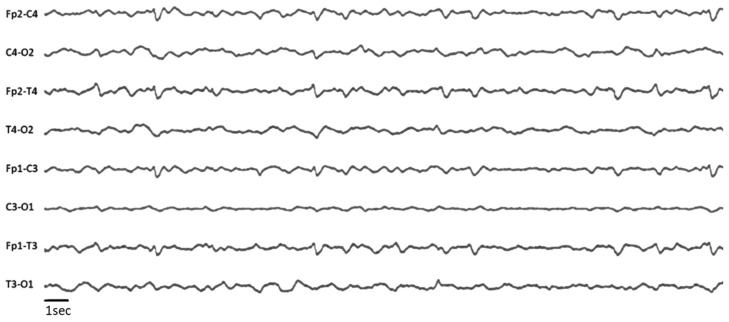
EEG recordings. 8 scalp electrodes, longitudinal bipolar montage, low frequency filter 0.53 Hz, high frequency filter 70 Hz, amplitude 100 μV/cm.

At days 55, 57, 63, and 65, repeated EEGs recorded diffuse and poorly reactive delta slow waves background activity, with periodic diphasic slow waves over both frontal areas (Figure 3). 

**Figure 4:**
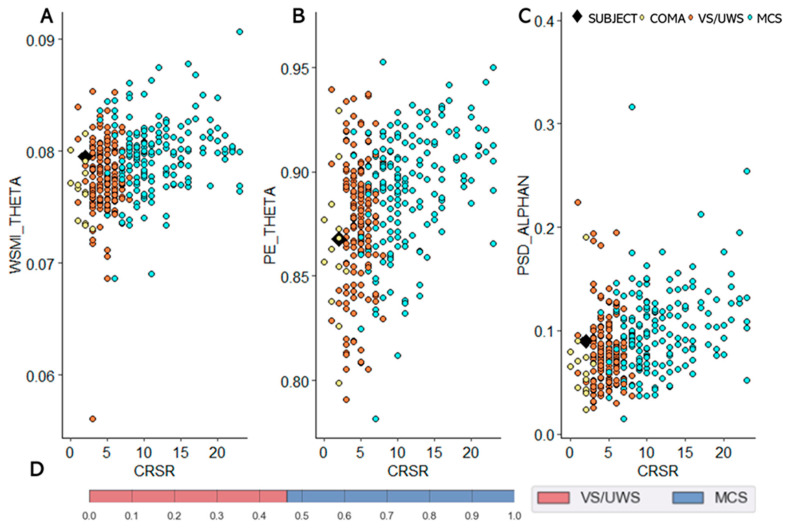
Quantitative EEG analysis. 28 EEG biomarkers (i.e. connectivity, spectral, information theory) are extracted from high-density EEG recordings during an auditory novelty task. Their combination within a support vector machine (SVM) algorithm allows to discriminate patients in a minimally conscious state (MCS) from those in a vegetative state that is also coined unresponsive wakefulness syndrome (VS/UWS) [19,20,21] Example of key EEG markers, (**A**): mean weight symbolic mutual information (WSMI) in theta band, (**B**): mean permutation entropy (PE) in theta band, (**C**): mean power spectral density (PSD) normalized in alpha band. Each plot displays one metrics (Y axis) in relation to CRS-r score (X axis). Patient is plotted in black square, VS/UWS patients in orange dots, MCS patients in blue dots, and comatose patients in yellow dots. (**D**): Result of the SVM classifier (Grid search). The patient was classified as MCS.

At day 55, somatosensory evoked potentials (SSEP) revealed a bilateral increase of P14-N20 inter-latencies). Brainstem auditory evoked potentials (BAEP) confirmed a brainstem impairment (right ear stimulation with low III and V wave amplitudes with increased intrapontine conduction time (I-V inter-latency), as well as left ear stimulation with no response at 110 dB stimulation level. At day 55 in spite of being clinically VS/UWS, the multivariate classification of high density EEG (hd-EEG) recordings, that uses spectral power, complexity and connectivity measures, was in favour of a minimally conscious state (MCS) [10,16,17] (Figure 4). Cognitive event-related potentials (ERP) using the “local-global” paradigm [18] showed neither mismatch negativity (MMN) nor P3b component.

**Figure 5:**
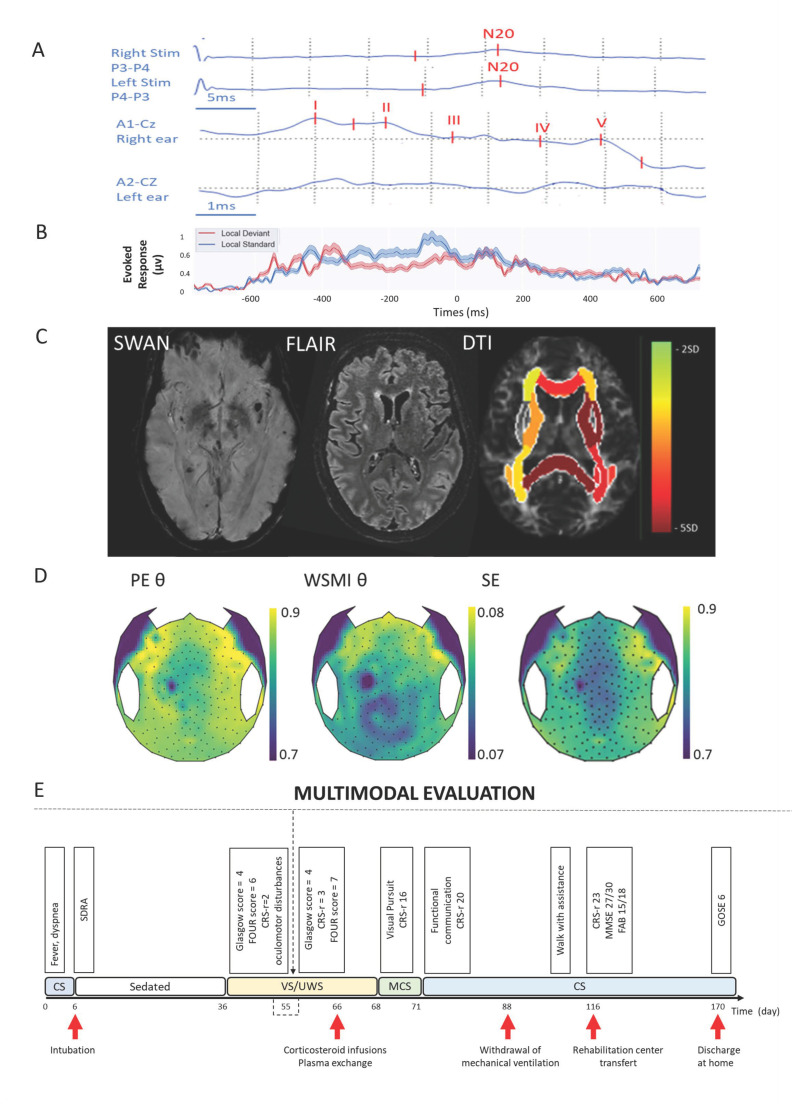
Illustration of the multimodal evaluation that combined SSEP and BEAP (**A**), brain qualitative and quantitative MRI (**B**), cognitive ERP (**C**), quantitative hd-EEG (**D**) and repeated clinical evaluations (**E**). (**A**) From top to bottom, left and right SSEP showing bilateral cortical responses (N20), left and right BEAP sowing brainstem impairment. (**B**) Brain MRI from left to the right, SWAN, FLAIR and DTI sequences. (**C**) local effect plot of the” local-global” paradigm; Neither mismatch negativity (MMN) or P3b component were presents. (**D**) Topographical plots of quantitative EEG biomarkers extracted from hd-EEG recording. From left to the right, weight symbolic mutual information (WSMI) in theta band, permutation entropy (PE) in theta band, spectral entropy (SE). (**E**) Longitudinal clinical follow-up of the patient’s recovery according to different scores assessing vigilance, consciousness and cognitive function together with pivotal behavior. CRS-r: Coma Recovery Scale Revised: CS, Conscious State; FAB: Frontal Assessment Battery; FOUR: Full Outline of Unresponsiveness GCS: Glasgow Coma Scale; GOSE: Glasgow outcome scale extended, hd-EEG: high density Electroencephalogram; MCS: Minimally Conscious State; MMSE: Minimental State Examination, SDRA: acute respiratory distress syndrome, VS/UW: unresponsive wakefulness syndrome.

At day 65, the patient was still unresponsive (FOUR-score = 7 (E2-M0-B4-R1), Glasgow coma scale = 4/15 (E2-V1-M1), (CRS-r) = 3/23 (1-1-0-0-0-1)). Given the suspected immune-related encephalitis, at day 66, we started, as a last resort, therapeutic option corticosteroid infusions (1g day IV methylprednisolone for 5 days) and therapeutic plasma exchange with albumin (5 sessions). A rapid and major improvement occurred from day 68, enabling functional communication at day 71. The withdrawal of mechanical ventilation could be achieved at day 88. A critical illness polyneuropathy was diagnosed. The patient was transferred to a rehabilitation center at day 116, with walking impairment (walk with two canes) and a mild dysexecutive syndrome (minimental state examination (MMSE) = 27/30, frontal assessment battery (FAB 15/18)). At 5.5 months, he was discharged home with only a mild attention deficit disorder, fatigability (walk with a cane for long distances) and a chronic cough (Glasgow outcome scale extended (GOSE) = 6), (Figure 5). Note that this patient has previously been reported in a case series focusing exclusively on therapeutic options in COVID-19 associated encephalitis [22].

## 3. Discussion

### 3.1. Disorders of Consciousness and COVID-19

When addressing impairments of consciousness within the context of COVID-19 infection, it is of tremendous importance to identify both the aetiology of brain damage and the type of DOC state ranging from a confusional state to minimally conscious state, vegetative state (also coined unresponsive wakefulness syndrome), and coma [19,20,21]. In the available studies the precise description of DOC type is not always documented. DOC prevalence varies across studies from 2.5% to 20% of patients hospitalized with COVID-19 [2,4,5,6,7,8,23,24] The prevalence of DOC is higher in older patients, with two or more comorbidities, and with severe COVID-19 infection [2,5,8].

Pathophysiological mechanisms underlying consciousness alterations during COVID-19 are highly variable and most likely multifactorial. Numerous of them are non-specific, related to sepsis, severe hypoxemia, toxic or metabolic encephalopathies in a context of multiorgan failure, ICU complications, and side effects of pharmacological treatment.

Loss of consciousness is also associated with cerebrovascular complications of COVID-19 [25], posterior reversible encephalopathy syndrome [26], as well as seizure and post ictal states [27]. The neurotropism of SARS-CoV-2 is highlighted by reports of acute necrotizing encephalopathy, meningitis, or encephalitis with SARS-CoV-RNA detection in the CSF [27,28]. In the present case study, an immune-related encephalitis was suspected, due to the association of a subacute onset of altered mental status, with focal neurological findings (oculomotor disturbance), MRI features suggestive of COVID-related encephalitis, and a reasonable exclusion of alternative causes (septic, metabolic and toxic encephalopathy, epileptic disorders, rheumatologic disorders etc.) [29]. Although we cannot rule out a spontaneous recovery, the impressive and rapid response to immunotherapies argues in favour of a dysimmune mechanism [22]. In our case, as in most patients with neurological manifestations, CSF analysis showed neither inflammation nor the presence of the virus [2,5,9]. COVID-19 related encephalitis and encephalopathy are still debated entities [30,31,32,33], and the underlying steps of SARS-CoV-2 immunopathogenesis have to be elucidated [34].

### 3.2. Consciousness Recovery Prediction in COVID-19 Related Encephalitis

#### 3.2.1. Time-Window for Neuroprognostication

The time-course of recovery of a patient with prolonged DOC after COVID-19 related encephalitis is largely unknown. The uncertainty about the underlying physiopathology and brain damages make difficult to identify the optimal time-window to determine neurological prognosis. On the one hand, a too early prognostication exposes to a risk of underestimation of patient’s clinical condition and potential of recovery, and to the corresponding inadequate decisions of withdrawal of life-sustaining therapies [35]. On the other hand, too late a prognostication may lead to therapeutic relentlessness. Our case highlights that the functional outcome can be unexpectedly good even in case of an initial catastrophic clinical presentation. More specifically, it also underlines that a full neurological recovery is still possible after two months of unresponsiveness in COVID-19 related encephalitis. Causal interpretation of the role of immunotherapy treatment is clearly beyond the scope of this single-case open label report, but we noted the patient’s fast improvement following a sustained period of poor functional state. 

#### 3.2.2. Clinical Evaluation 

Despite medical technology advances, clinical examination remains crucial in neurological prognostication. Clinical signs (absence of pupillary and corneal reflexes, no motor response to painful stimuli, myoclonic status epilepticus) and behavioural scores (Glasgow coma scale, full outline of unresponsiveness score, coma recovery scale revised) are currently used for their prognostic accuracy in ICU [36,37,38]. Their prognostic value is questionable in COVID-19 related encephalitis. Cases with unexpected good outcome despite catastrophic initial presentation have been reported [1,39]. In this case, for two months repeated behavioural scales and clinical assessment of brainstem stayed alarming, and yet at six months the patient only presented minor neurological and psychological deficits. 

#### 3.2.3. Brain Imagery 

Reports of neuroimaging findings in patients with severe COVID-19 are growing, but only few data are available concerning their prognostic values in DOC [3,40,41,42,43,44]. Neuroradiological features are variable, dominated by acute ischemic infarcts and haemorrhagic lesions but also included non-confluent multifocal WM hyperintense lesions on FLAIR and diffusion with variable enhancement, perfusion abnormalities, deep venous thrombosis, and posterior reversible encephalopathy syndrome [3,40,43]. Among them, markers of poor outcome published are mainly acute large ischemic stroke or haemorrhagic stroke [41]. After the exclusion of ischemic infarcts and cerebral venous thrombosis, patients with haemorrhagic lesions also seem to have more severe clinical presentation [4]. 

In COVID-19 patients, basal ganglia, cortical, and white matter lesions have been reported without assessing the relationship of brain lesion localisation to the outcome [3,40,44]. In the present case, widespread white matter tracts, cortical, subcortical, and pontin regions were damaged. Interestingly some lesions were localised on regions identified as a potential key neural structure necessary to awareness, the posterior brainstem that includes the ascending reticular system and the subinsular regions that includes the claustrum, a narrow grey matter structure massively connected to distant associative cortices [45,46,47]. Here, brainstem dysfunction was confirmed both by an alteration of the brainstem auditory evoked potentials and by clinical evidence (skew deviation and left internuclear ophthalmoplegia). Brainstem lesions in association with COVID-19 have been reported as rhombencephalitis [39,48], acute necrotizing encephalopathy [49,50], and central pontine myelinosis [40]. Moreover, brainstem dysfunction may be involved in the respiratory failure of COVID-19 patients [50,51]. 

Promising neuroimaging techniques enable the study of network structural connectivity, to improve outcome prediction of patients with brain injury. In a similar COVID-19 related encephalitis case, the demonstration of intact network connectivity with functional MRI helped to prognostic a good outcome [1]. In our case, structural connectivity, assessed with quantitative analysis of white matter injuries estimated with DTI-MRI was equivocal. FA was decreased with an asymmetrical pattern for left prevalence. Based on post-anoxic normative values, whole-brain white matter fractional anisotropy (WWM-FA) was in an uncertain ‘grey zone’ for outcome prediction: patient’s value was above a threshold associated with an unfavourable outcome, but below a threshold associated with a favourable outcome [15]. However, the decreased FA within the corpus callosum suggested a poor neurological prognosis. 

#### 3.2.4. Clinical EEG

EEG is commonly used in the evaluation of patients with DOC for etiological diagnosis (e.g., seizures and post ictal states, acute viral encephalitis, metabolic or toxic encephalopathies) as well as for prognostication. As previously described in patients with COVID-19, we could also observe a non-specific diffuse slowing of background activity [3,32,52], as well as bifrontal monomorphic periodic delta waves [53,54]. Periodic and rhythmic discharges have been associated with poor outcomes in several series of critically ill patients [55,56,57]. However, in COVID-19 patients, with similar periodic EEG patterns, opposite outcomes have been reported, suggesting that these EEG figures are not predictive of neurological outcome [54].

Absence of reactivity to external stimulation is usually considered as a bad outcome marker, in particular in post-acute severe brain injury [58,59,60]. In COVID-19 patients, background reactivity when reported, was absent or weak and was not correlated with the neurological prognosis [3,32,52]. In our case, the patient recovered consciousness in spite of an initial poor EEG reactivity to external stimulations.

#### 3.2.5. Evoked Potentials 

Evoked potentials are more and more commonly used in critically ill patients. In response to sensory, auditory, or visual stimuli, early and middle latency responses evaluate the functional integrity of brainstem and cortical modules. In post anoxic comatose patients, the loss of cortical responses in somatosensory evoked potentials (SSEP) and the loss of bulbo-protuberantial responses in brainstem evoked potentials (BEAP) are highly predictive of poor neurologic outcome defined as death or survival in vegetative state [61,62,63]. However, the use of SSEP and BEAP for assessing consciousness and discriminating a vegetative state from a minimally conscious state is limited. Some level of residual cognitive processing can be revealed, with more delayed and integrated components in response to standard and deviant stimuli. Cerebral responses to violations of temporal regularities that are either local in time or global across several seconds reflect, respectively, either an automatic detection of auditory violation (mismatch negativity) or a conscious processing of the auditory environment (P300) [18]. Our case highlights the limit of evoked potentials. SSEP cortical responses were delayed but present. In the context of sepsis and multiple organ failure, the intracranial and intrapontine conduction times explored with SSEP have been demonstrated to be prolonged in patients compared to controls, but without establishing a relationship with the outcome [64,65,66]. Because of the major alteration of BEAP, cognitive event-related potentials using the “local-global” paradigm were uninterpretable.

#### 3.2.6. Quantitative EEG 

Quantitative analysis of hd-EEG in our patient was more congruent with his outcome. Based on the combination of 28 EEG biomarkers from four conceptual families (i.e. connectivity, spectral, information theory, and evoked responses) the patient was automatically classified as being in a minimally conscious state by a support vector machine classifier [10,17] in spite of being clinically VS/UWS during hd-EEG recording. One of the assets of this approach to predicting consciousness recovery consists in pooling together all possible aetiologies of DOC in a large EEG database to train the SVM algorithm [10,11,17,67]. It may have captured some key functional neural properties specific to consciousness, irrespective of the lesional mechanism at work. Statistically, the degree of confidence of the SVM classifier was moderate (area under the curve (AUC) = 0.76, predicted probabilities: MCS = 54% compared to VS/UWS = 46%). However, within multimodal evaluation, this good prognostic factor combined with equivocal MRI DTI data allowed for a degree of uncertainty which motivated continuing maximum care and treatment.

## 4. Conclusions

In order to more accurately predict the recovery of consciousness in DOC patients suffering from COVID-19 related encephalitis, we propose to take advantage of the multimodal approach exposed in the present case report with a special emphasis on multimodal EEG algorithms that could classify the patient as MCS early on. Future long-term outcome studies should identify the most relevant prognostic markers for this specific aetiology of DOC and guide potential treatment.

## Supplementary Materials

The following are available online at https://www.mdpi.com/2076-3425/10/11/845/s1. File S1: CoCo-Neurosciences Study Group: List of Affiliations for Publications.
